# Supplementary material for: Solventless Photopolymerizable Paper Coating Formulation for Packaging Applications
Source: Polymers (Basel). 2023 Feb 21;15(5):1069. doi: 10.3390/polym15051069 (PMC10005472; doi:10.3390/polym15051069)
Supplement: Supplementary file 1 [file polymers-15-01069-s001.zip › polymers-2194966-supplementary.pdf]

polymers

Supplementary Information

Solventless Photopolymerizable Paper Coating Formulation for Packaging Applications

Fábio M. Silva, Ricardo J. B. Pinto, Ana Barros-Timmons and Carmen S. R. Freire *

CICECO—Aveiro Institute of Materials, Chemistry Department, University of Aveiro, 3810-193 Aveiro, Portugal
* Correspondence: cfreire@ua.pt

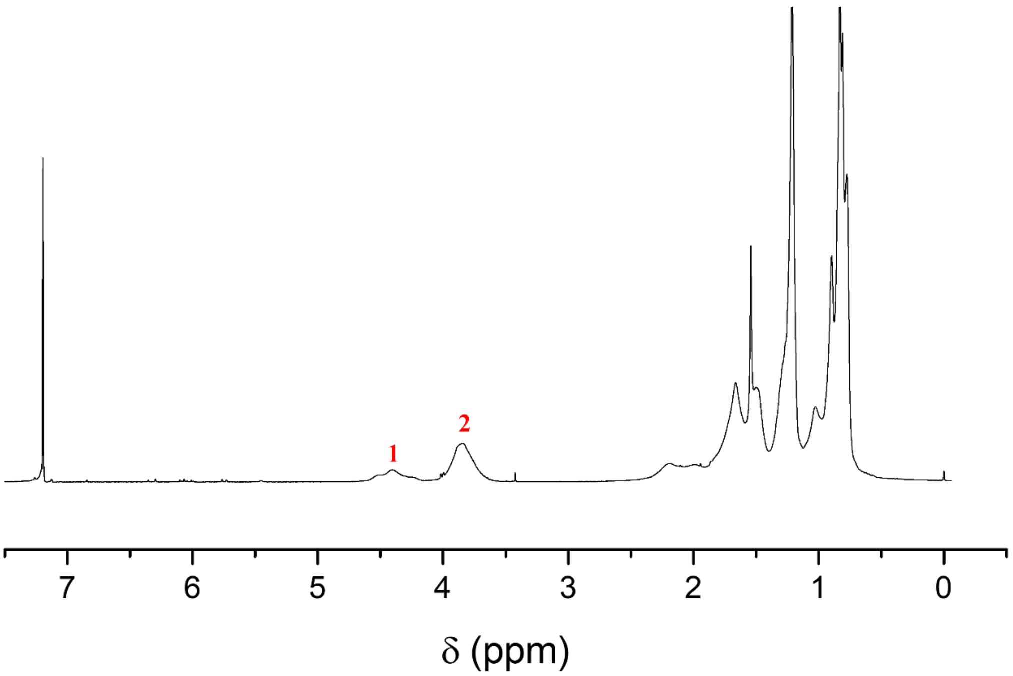

Figure S1. ^{1}H NMR spectra of the copolymer poly(2-ethylhexyl acrylate-co-isobornyl methacrylate) after purification. The chemical shifts identified with numbers 1 and 2 (in red), at 4.40 and 3.84 ppm, are assigned to the hydrogen of the ortho-carbon of the isobornyl methacrylate's ring and to the two hydrogens of the methylene group linked to the oxygen of the ester group of 2-ethylhexyl acrylate, respectively.
